# A Serendipitous Histopathological Revelation: An Intriguing Case Report of a Cystic Schwannoma of the Lateral Neck

**DOI:** 10.7759/cureus.83515

**Published:** 2025-05-05

**Authors:** Ravindran Chirukandath, Tinu Sasi, Hyfa Anan, Arya Rajesh Nair, Saranya Sasidharan

**Affiliations:** 1 General Surgery, Government Medical College, Thrissur, IND

**Keywords:** branchial cyst, cystic degeneration, cystic neck swelling, rare head and neck, schwanoma

## Abstract

Schwannomas are rare, benign tumors that arise from Schwann cells, which produce the myelin sheath around peripheral nerves. Cystic degeneration is an uncommon feature of schwannomas, occurring in approximately 4% of cases. The diagnosis of cystic schwannomas can be challenging due to their rarity and nonspecific clinical and radiological features.

A 45-year-old female presented with a two-year history of a gradually enlarging neck mass, which was initially suspected to be a branchial cleft cyst. Computed tomography (CT) scans showed a well-defined cystic lesion in the left supraclavicular region. The patient underwent ultrasound-guided fine-needle aspiration cytology (FNAC), which yielded hypocellular fluid. The patient underwent surgical excision of the mass, which was subsequently diagnosed as a cystic schwannoma based on histopathological examination.

Cystic schwannomas can pose a diagnostic challenge due to their rarity and nonspecific clinical and radiological features. Awareness of the clinical and pathological features of cystic schwannomas is essential for timely diagnosis and effective treatment. Accurate diagnosis is crucial to ensure optimal management and prevent recurrence.

## Introduction

Schwannomas, also referred to as neurilemmomas, represent rare, benign neoplasms originating from Schwann cells, the neuroectodermal elements responsible for the elaboration of the myelin sheath enveloping peripheral nerves. These tumors are characteristically solitary, well-circumscribed, and encapsulated, often demonstrating a distinct cleavage plane from adjacent anatomical structures. They exhibit a predilection for the head and neck region and the flexor surfaces of the extremities, with approximately one-third manifesting within the cephalocervical domain. Among these, the parapharyngeal space constitutes the most frequently involved site [1].

On cytological examination, schwannomas typically exhibit both Antoni type A and type B tissue patterns. The cytological features generally mirror the histopathology, with Antoni type A areas showing compact, cohesive fascicles of spindle-shaped cells with varying cellularity. These regions often contain dense fibrillary stroma, palisading nuclei, and structures resembling Verocay bodies. The individual tumor cells appear as cohesive spindle or oval cells with tapering ends and indistinct cytoplasmic borders, along with noticeable variability in nuclear size and shape. Antoni type B areas, in contrast, demonstrate loosely arranged, wavy spindle cells dispersed in a myxoid matrix, often accompanied by microcyst formation, histiocytes, and lymphocytes. Immunohistochemical staining for S-100 protein typically shows strong, diffuse positivity. Although schwannomas predominantly exhibit a solid architecture, cystic degeneration is an infrequent histopathological feature, documented in merely 4% of cases [2]. The diagnosis of such lesions remains inherently challenging due to their relative infrequency and the limited diagnostic yield of fine needle aspiration cytology (FNAC) in this context, a modality otherwise instrumental in the evaluation of more common cervical masses.

Of particular diagnostic concern is the potential for schwannomas exhibiting cystic transformation to simulate branchial cleft cysts, both clinically and radiologically. This distinction is of paramount importance, as branchial cysts are prone to recurrence and necessitate long-term surveillance, whereas complete surgical excision of a schwannoma is typically curative, with recurrence being exceptionally rare.

Herein, we delineate the case of a 45-year-old female who presented with a left-sided cervical mass, subsequently identified as a schwannoma with prominent cystic degeneration, underscoring the necessity for heightened clinical vigilance and thorough histopathological evaluation in such diagnostically ambiguous presentations.

## Case presentation

A 45-year-old female presented with a swelling on the left side of her neck, which had been gradually increasing in size over the past two years, with a noticeable increase over the last four months. The swelling was associated with pain, but there were no complaints of dysphagia or breathing difficulty. On clinical examination, a 3×3 cm, mildly tender swelling was noted in the left supraclavicular region, located in the posterior triangle of the neck. The mass had well-defined borders, was firm in consistency, mobile, and not fixed to underlying muscles.

Initially, USG of the neck (Doppler) was done on the 25th of January, 2024, which showed colloid nodules in both lobes of thyroid had Thyroid Imaging Reporting and Data System (TI-RADS) TR 2 lesions, and a cystic lesion in the left supraclavicular region with a possibility of branchial cleft cyst or lymphangioma.

**Figure 1:**
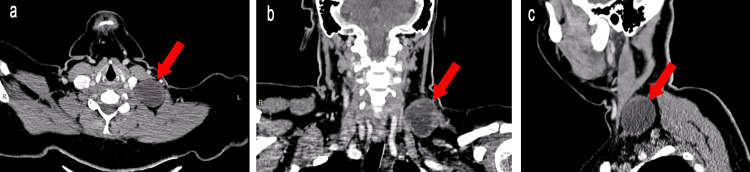
Contrast CT of neck a) Axial view showing a well-defined cystic lesion on the left side abutting the carotid vessel; b) Sagittal view showing the lesion abutting the paraspinal muscles; c) Coronal view showing a lesion at the root of the neck

CT neck (plain and contrast study) showed a well-defined cystic lesion in the left side of rgw neck, with a few enhancing soft tissue densities in the peripheries, and the suggested possibilities were lymphangioma, cystic lymph node, and third branchial cleft cyst (Figure 1).

**Figure 2:**
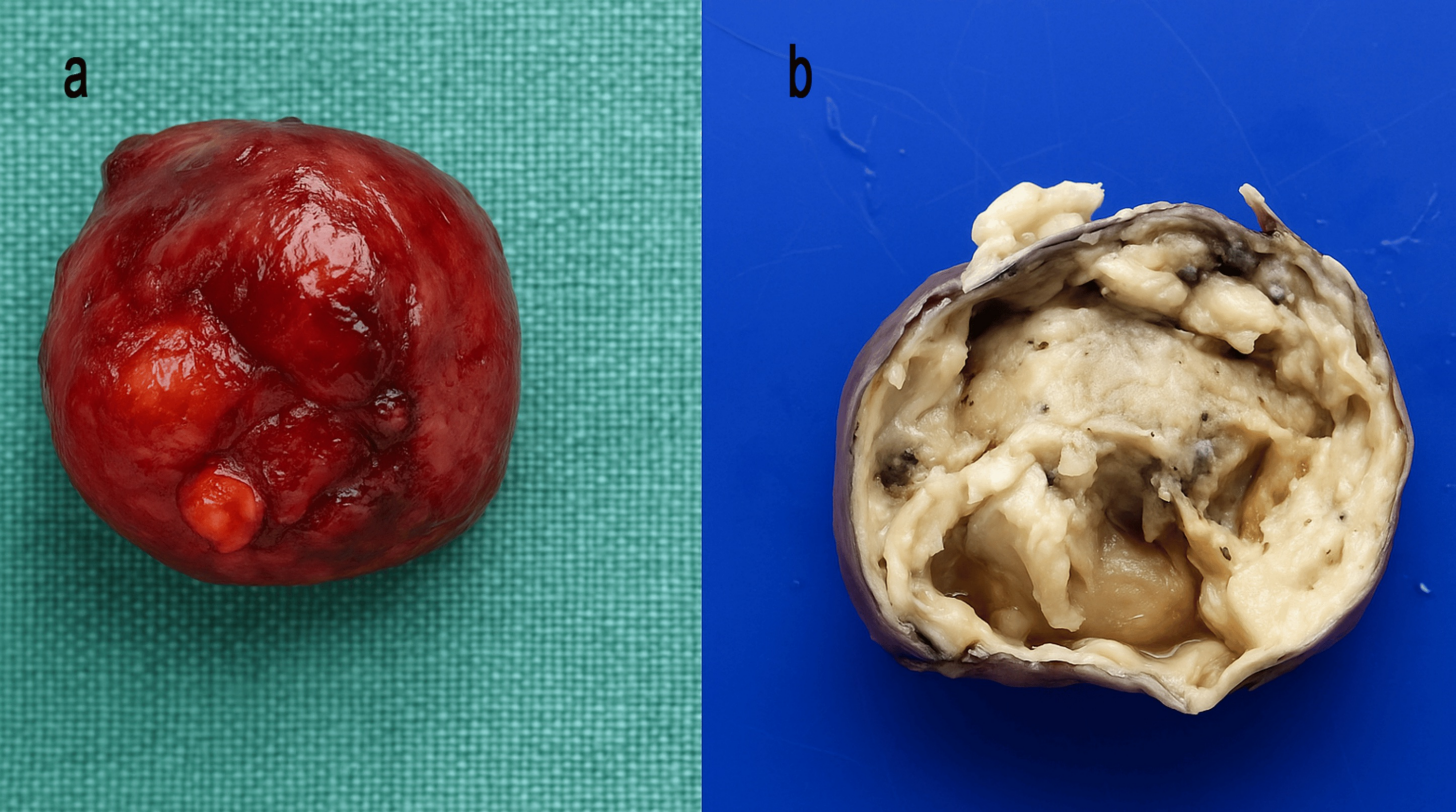
Gross morphology a) Cyst immediately after excision; b) Cut section of the lesion showing cystic spaces

Further evaluation with FNAC of the swelling left supraclavicular region, repeated attempts aspirated reddish fluid, and smears show only scattered macrophages, no epithelial cells seen in the smear, suggestive of a cystic lesion. The patient was taken up for surgery. A lateral neck cystic swelling was excised in toto under general anaesthesia (Figure 2).

**Figure 3:**
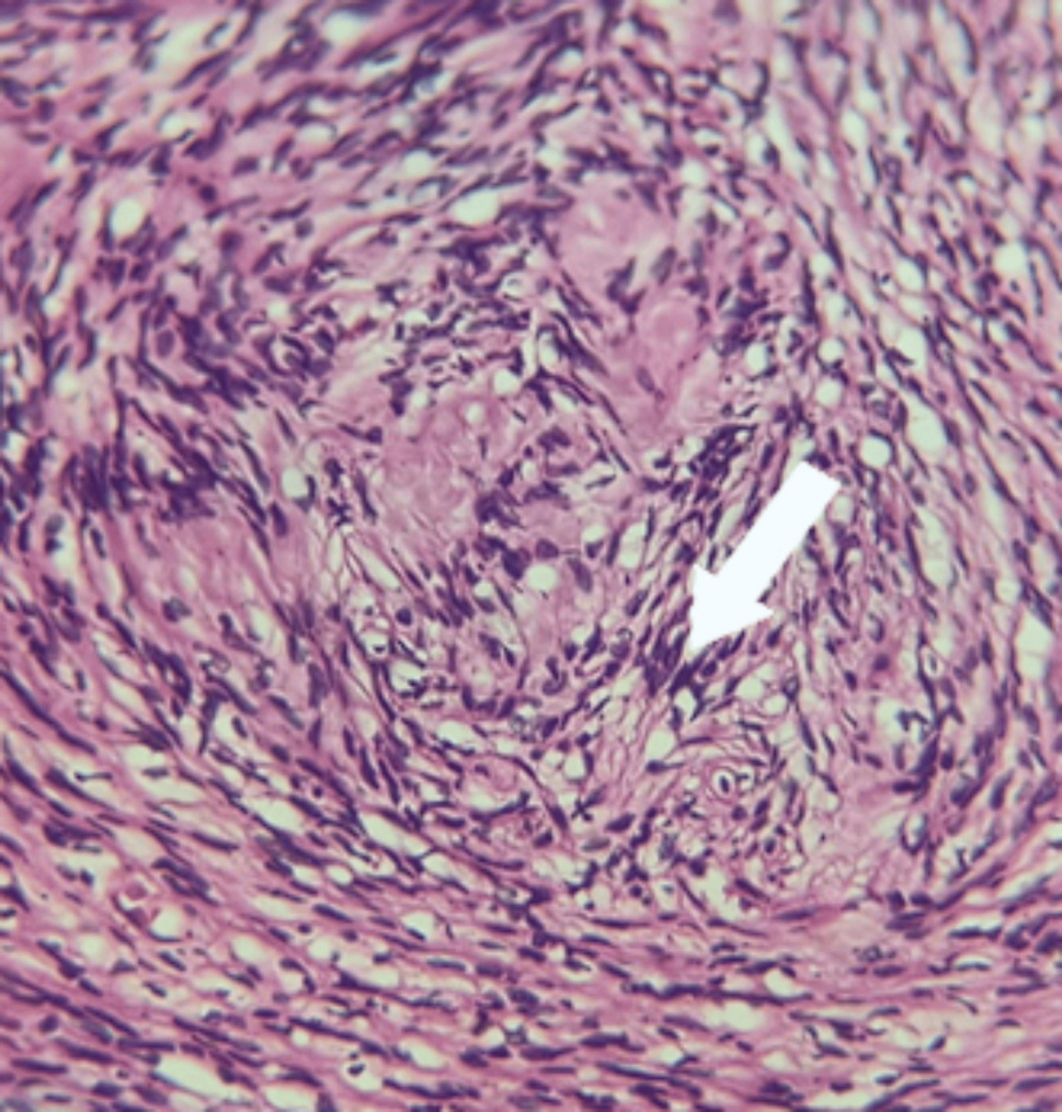
Histopathology showing hypercellular areas

Histopathology revealed a neoplasm composed of hypercellular and hypocellular areas. Individual cells are narrow, elongated, and wavy with a tapered end, interspersed with collagen fibers, nuclear palisading forming Verocay bodies noted, mitosis infrequent, and finally diagnosed to be Schwannoma (Figure 3).

## Discussion

Schwannomas are benign neurogenic neoplasms arising from Schwann cells, the glial elements responsible for forming the myelin sheath of peripheral nerves. Approximately one-third of these tumors occur within the head and neck region, where they may originate from cranial, peripheral, or autonomic nerves [3]. Among the cranial nerves, the eighth cranial nerve (vestibulocochlear) is most commonly affected. The identification of the nerve of origin in schwannomas can be challenging, with approximately 10-40% of cases remaining indeterminate despite radiological and pathological evaluation [4]. This uncertainty complicates diagnostic efforts, as many of the nerves in the head and neck are small and unnamed, making it difficult to pinpoint the precise origin of the tumor. In this case, the tumor's presentation in the posterior triangle of the neck, where cystic schwannomas are rare, along with the lack of neurological symptoms, added complexity to the diagnosis. The lesion initially appeared to be a benign cystic mass, leading to a delayed recognition of its true nature. This case is noteworthy due to the unusual location of the schwannoma and its atypical presentation, highlighting the importance of considering a broader differential diagnosis and utilizing advanced imaging and pathological tests for accurate diagnosis.

Schwannomas arising in the parapharyngeal space often present as asymptomatic, slow-growing cervical masses along the medial border of the sternocleidomastoid muscle. Clinical diagnosis is frequently delayed due to the indolent nature of these lesions. When symptomatic, hoarseness and paroxysmal cough elicited by palpation may serve as subtle clinical indicators. The differential diagnosis is broad, encompassing branchial cleft cysts, paragangliomas, and malignant lymphomas [5,6].

A subset of schwannomas undergo cystic degeneration, a phenomenon observed in approximately 4% of cases [7,8]. These cystic changes complicate preoperative diagnosis, as they are associated with rapid tumor enlargement and increased lesion size. The pathogenesis of cystic degeneration is attributed to necrosis, mucinous degeneration, hemorrhage, and microcyst formation, particularly within Antoni B areas, which are characterized by loosely arranged spindle cells.

Fine-needle aspiration cytology (FNAC) often yields hypocellular or hemorrhagic aspirates in cases of cystic schwannoma, thereby limiting its diagnostic utility. The rarity of these lesions, combined with the cytological paucity, renders FNAC alone inadequate for definitive diagnosis. However, ultrasound-guided FNAC may enhance diagnostic accuracy by enabling targeted sampling from hypercellular Antoni A regions, thereby increasing the probability of capturing diagnostic tissue.

Preoperative assessment of schwannomas relies heavily on advanced radiological imaging, with magnetic resonance imaging (MRI) being the modality of choice. MRI provides critical information regarding tumor localization, extent, and relationship to adjacent vascular structures such as the jugular vein and carotid artery. Despite radiological insights, definitive diagnosis hinges on histopathological examination, which classically reveals a biphasic pattern comprising Antoni A and Antoni B areas, palisading spindle cells, and the presence of Verocay bodies. Immunohistochemical staining, particularly diffuse S100 protein positivity, further substantiates the Schwann cell origin of these neoplasms [9].

Occasionally, lymphoid aggregates and scattered lymphocytes may be identified within schwannomas, adding to the diagnostic complexity and mimicking cystic lymphoid lesions. Therefore, in the presence of a cystic lesion with lymphoid components, cystic schwannoma should be considered in the differential diagnosis to avoid misclassification [10].

Accurate distinction between branchial cleft cysts and schwannomas is of paramount importance, given their differing clinical courses and management strategies. Incomplete excision of a branchial cleft cyst predisposes to recurrence, necessitating long-term surveillance. Conversely, complete resection of a schwannoma is typically curative, with recurrence being rare. While surgical excision remains the standard of care, conservative management may be judiciously employed in select, asymptomatic cases [11,12].

## Conclusions

In conclusion, schwannomas with cystic degeneration can pose a diagnostic challenge due to their rarity and nonspecific clinical and radiological features. A high index of suspicion and careful histopathological examination are essential to distinguish these tumors from other cystic lesions, such as branchial cleft cysts. Complete surgical excision is the treatment of choice for schwannomas, and accurate diagnosis is crucial to ensure optimal management and prevent recurrence. Awareness of the clinical and pathological features of cystic schwannomas can aid in timely diagnosis and effective treatment.
